# Prediction of pathological fracture in patients with metastatic disease of the lower limb

**DOI:** 10.1038/s41598-019-50636-9

**Published:** 2019-10-01

**Authors:** Emma L. Howard, Paul Cool, Gillian L. Cribb

**Affiliations:** 1 Robert Jones and Agnes Hunt Orthopaedic Hospital NHS Foundation Trust, Gobowen, SY10 7A UK; 2 School of Medicine, Keele University, Keele, ST5 5BG UK; 3 University of Manchester, Oxford Road, Manchester, M13 9PL UK

**Keywords:** Outcomes research, Surgical oncology, Risk factors

## Abstract

The aim of this study was to investigate if the risk of pathological fracture can be predicted with the proportion of body weight that can be put through the affected leg in patients with metastatic bone disease of the lower limb. A prospective observational study was conducted in patients with metastatic disease in the lower limb. Receiver Operator Characteristic curves were used to identify the optimum threshold level of single stance weight bearing to predict fracture and compared to the Mirels score. Patients who underwent surgery could weight bear significantly less than those who did not have surgical intervention. The optimum threshold to predict pathological fracture was 85% of total body weight. No patient below the threshold level of 85% single stance body weight sustained a pathological fracture. The use of single stance body weight can be a useful in conjunction with the Mirels score to predict pathological fracture. If less than 85% of total body weight can be put through the affected limb, the risk of fracture increases, and consideration of treatment is suggested.

## Introduction

Pathological fractures which arise as a consequence of metastatic disease compromise patient morbidity and mortality^1,2^. They are painful and affect mobility. Pathological fractures require urgent clinical evaluation and usually surgery is performed in less than ideal conditions^1^. The mainstay of clinical management for metastatic bone disease is palliative and aims to improve quality of life, avoiding pathological fracture^3,4^.

The literature reports that patients undergoing prophylactic fixation of metastatic lesions lose less blood in theatre, have a shorter inpatient stay and superior functioning after surgery than patients who undergo fixation of a pathological fracture^5^. Prophylactic fixation is also reported to prevent the complications of pathological fractures, namely delayed or non-union and the need for re-operation^6^.

Consequently, it is of importance that clinicians can accurately predict impending pathological fractures or be reassured when fracture is unlikely. This ensures that prophylactic fixation is only performed when necessary, and when the benefits of fixation outweigh the risks of major surgery.

Mirels developed a scoring system to predict impending fractures. The score has four components: anatomical site, size, radiographic appearance of the lesion and severity of pain. Each component can be scored from one to three and consequently the Mirels score ranges from four to 12. A Mirels score of nine estimates a 33% risk of pathological fracture and surgical intervention is recommended^7^. Although the Mirels score is commonly used in clinical practice, it has several limitations as most of the components of the score are subjective and prone to variation^8^.

The purpose of this study was to evaluate if the proportion of body weight that can be put through the affected leg can predict pathological fractures in patients with metastatic disease of the lower limb and to compare this to the Mirels score.

## Patients and Methods

A prospective study approved by the Health Research Authority (HRA: 233366) was conducted on all adult patients with metastatic disease of the lower limb presenting to a specialist bone tumour unit between September 2013 and September 2017. It was agreed by the HRA that data was collected as part of routine clinical evaluation and that explicit written consent was not required.

Patients were excluded if they had less than three months radiological follow up, a pelvic lesion, a primary bone tumour or were unable to stand to have their body mass measured using hospital weighing scales. Radiological follow up duration of three months was deemed to be sufficient to predict impending pathological fractures.

All patients had their single stance weight measured on their affected and unaffected leg by an independent hospital practitioner using a hospital weighing scale (Marsden M-430). Patients were requested to stand with both legs on the weighing scale and the total body weight was recorded. Firstly, the patient was asked to raise the affected leg and the maximum amount of body weight the unaffected leg could bear was read from the weighing scale. Subsequently, the maximum amount of body weight the affected leg could bear was measured by asking the patient to raise the unaffected leg. Patients could use crutches or aids for support if so required. The proportion of body weight that could be put through each leg was calculated and expressed as a percentage of total body weight. Every patient had a Mirels score by one of the senior authors. Data on Body Mass Index (BMI), patient outcomes and management were also collected. Based upon full clinical evaluation, the senior authors judged the patient’s fracture risk as either high or low. This risk assessment was based on the amount of pain the patient perceived, the bone involved (femur, tibia or fibula), radiographic appearances (lytic, mixed or sclerotic), size of the lesion and location of the lesion within the bone (i.e. a medial lesion in the proximal femur is at higher risk of fracture than a lateral lesion of the same size and appearances). Where appropriate, patients also had computerised tomography scans and magnetic resonance imaging to assess fracture risk. Features of these investigations, such as the amount of oedema, were also included in the overall risk assessment. All patients were discussed between the two senior authors as part of the evaluation and classified as either high or low risk. The proportion of body weight that could be put through the affected leg was not available to the senior authors at time of classification.

### Statistical analysis

Data were analysed with R statistical software (R Foundation, Vienna, Austria). Receiver Operator Characteristic (ROC) curves were produced to compare the proportion of weight that could be put through the affected leg with the Mirels score. The threshold level of single stance weight which could be used to indicate impending pathological fracture was determined from the ROC curve^9^. The value with the highest correct prediction and lowest false positive rate was identified from the curve. The diagnostic accuracy of the proportion of body weight that the affected leg could bear was also calculated and compared to the Mirels score^10^. Data could not be modelled with a normal distribution and Wilcoxon rank sum tests were used for statistical analysis. A p-value of less than 5% was considered significant.

## Results

Initially, 73 patients were identified from the database. Fourteen patients were excluded from the study as they had a pelvic lesion, an actual pathological fracture, or had surgery for a non-oncological reason. Of the 59 patients who met the inclusion criteria, 31 patients (53%) were female and 28 patients (47%) were male. The primary malignancy originated from the breast in 26 (44%) patients, prostate in 13 (22%) patients, kidney in seven (12%) and lung in four (7%) of patients. One patient (2%) had metastatic carcinoma of the lacrimal gland, two patients (3%) had multiple myeloma, one patient (2%) had lymphoma of bone, and five patients (8%) had metastatic carcinoma of unknown primary. 53 (90%) of lesions were located in the femur, four (7%) in the tibia and two (3%) were located in the fibula. The mean age was 65 years (range 35 to 89).

The mean single stance weight patients could put through their affected and unaffected limb was 84% (range 0–100%) and 94% (range 0–102%) of total body weight respectively. The mean Body Mass Index (BMI) of all patients was 27 (range 15–42). The mean length of follow-up was 0.95 years (range 0.25–4.42 years).

Twenty-six patients (44%) were deemed to have a high fracture risk and 33 patients (56%) were considered to have a low risk of fracture. In the high-risk group, 18 patients (70%) underwent surgery, four patients (15%) declined surgical management (due to limited pain), and four patients (15%) were deemed unfit for surgery. The four patients who declined surgical treatment all could bear more than 96% of body weight through the affected leg. None of these patients sustained a subsequent pathological fracture. Of the four patients who were deemed unfit for surgical treatment, all could bear only limited weight through the affected leg (58–90%) and had a very poor mobility. Three of these patients died after a short follow up, but did not sustain a pathological fracture. The remaining patient survived for over a year, but was bed bound with very poor general health.

**Figure 1:**
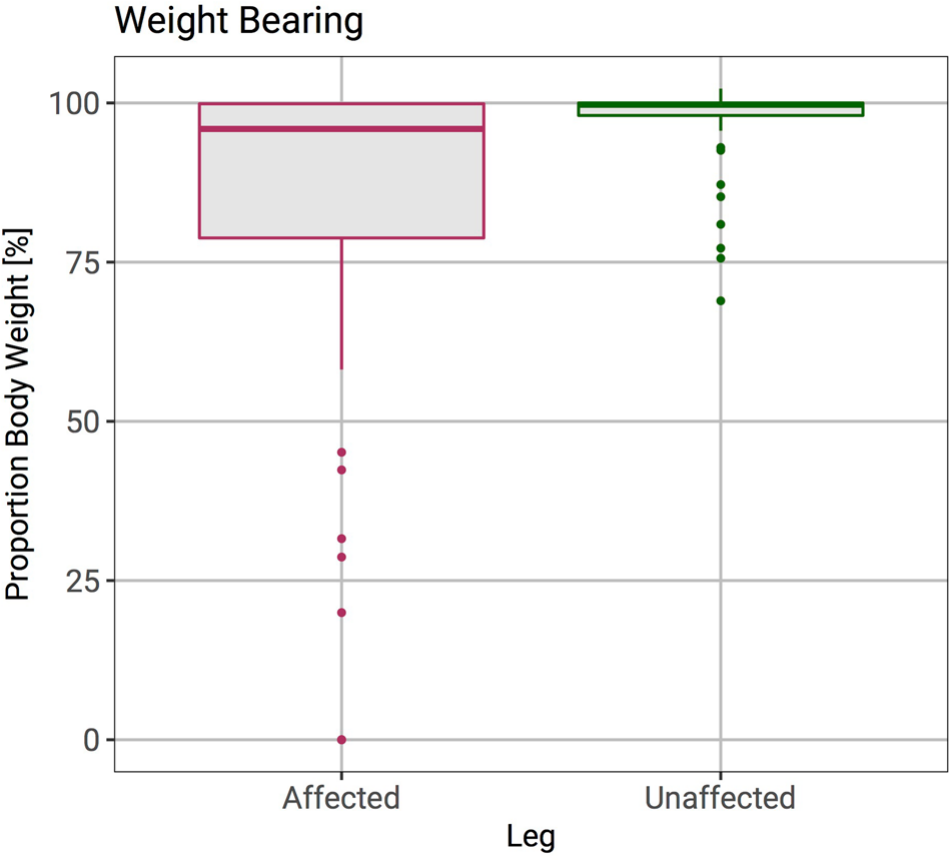
Box plots showing the proportion of total body weight that the affected and unaffected leg could bear. Weight bearing in the affected leg is significantly reduced and has a greater variance.

In the low risk group, 30 patients (91%) were not offered surgical management, two patients (6%) were deemed unfit for surgical intervention and one patient (3%) underwent surgery. The patient in the low risk group who had surgical intervention had complete excision of a solitary breast metastasis with curative intent. No patients in the low risk group sustained a subsequent pathological fracture. Figure 1 compares the proportion of total body weight patients could put through their affected and unaffected limb. There is more variance in the proportion of weight bearing in the affected limb than the unaffected limb. A Wilcoxon rank sum test showed the difference in proportion of body weight bearing between the affected and unaffected leg to be statistically significant (p < 0.001).

**Figure 2:**
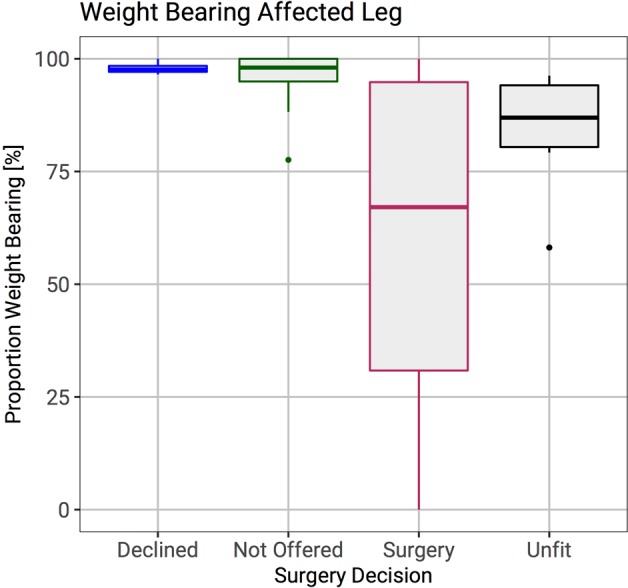
Box plots showing the ability to weight bear on the affected leg grouped by surgical decision. Weight bearing in the affected leg was significantly reduced in patients who subsequently had surgical intervention.

**Figure 3:**
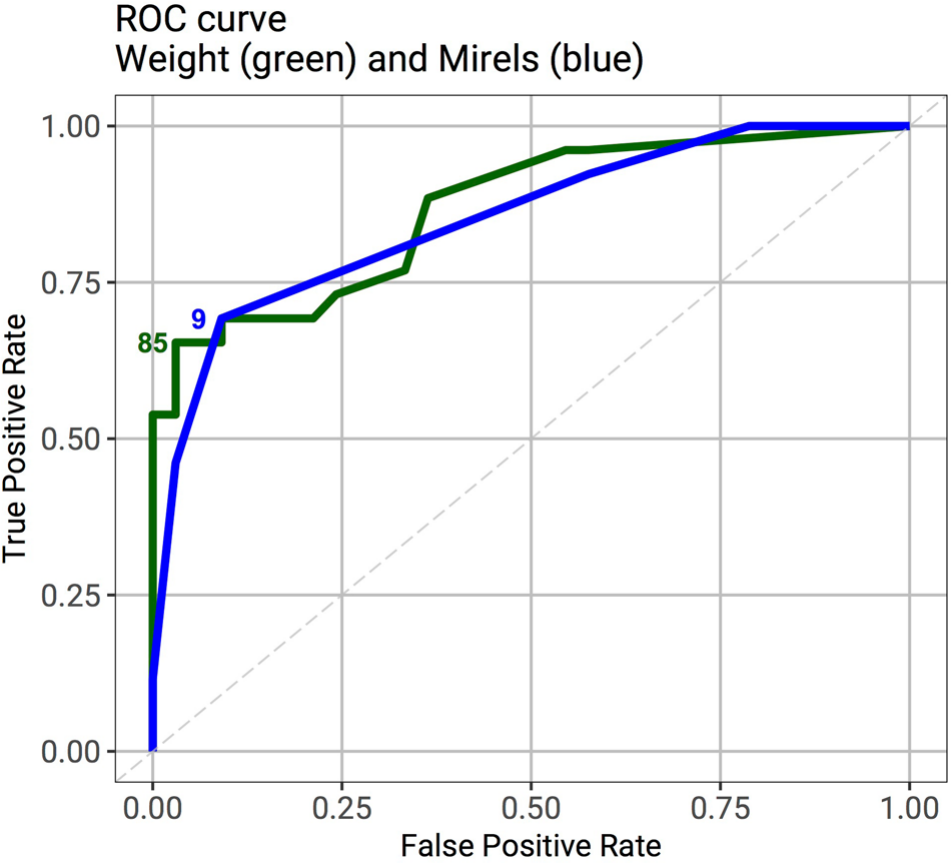
ROC curves comparing the Mirels score to the proportion of body weight that can be put through the affected leg. The area under the curve for the proportion of weight that can be put through the affected leg (87%) is similar than for the Mirels score (85%). In agreement with Mirels’ paper, a Mirels score of nine is the optimal classifier to predict impending pathological fracture. The optimal classifier for pathological fracture for the proportion of weight bearing through the affected leg is 85%.

Patients who underwent surgery could put less weight through their affected limb than patients who were not offered surgery. Interestingly, patients who declined surgical management could bear a high proportion of their body weight through the affected leg (Fig. 2). The proportion of weight bearing through the affected leg was significantly lower in patients who were classed as high fracture risk than those who were considered to be at low risk of fracture (Wilcoxon rank sum test; p < 0.001). Receiver Operator Curves (ROC) were compared for the Mirels score and proportion of body weight through the affected leg (Fig. 3). The area under the curve was 85% for the Mirels score and 87% for the proportion of body weight that could be put through the affected leg. When comparing the ROC curves, a Mirels score of nine and single stance body weight of 85% through the affected leg were the optimum classifiers.

**Figure 4:**
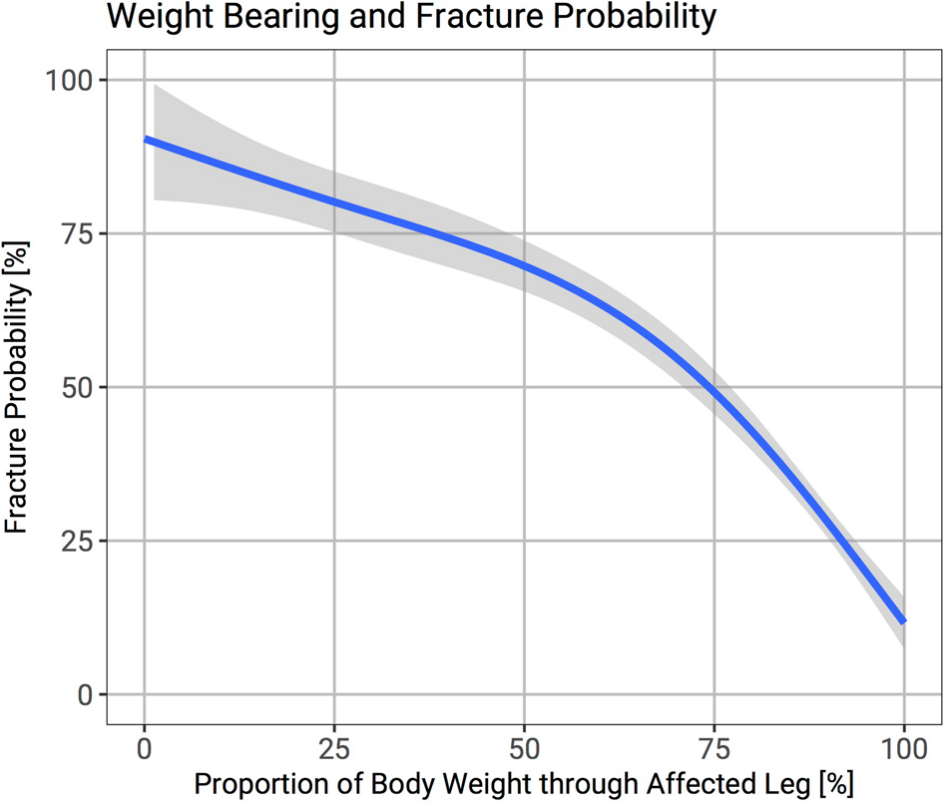
Probability of fracture as the proportion of body weight patients can put through their affected leg changes. The shaded grey area represents the 95% confidence interval.

A fracture probability curve illustrates how the risk of fracture changes with the proportion of body weight that could be put through the affected leg (Fig. 4). The sensitivity, specificity, positive predictive value, negative predictive value, and accuracy of the proportion of body weight that could be put through the affected limb (threshold 85%) compared to the Mirels score (threshold 9) are shown in Table 1.

**Table 1:** Comparison of the classifiers for the Mirels score (nine) and proportion of weight bearing through the affected leg (85%).

	Mirels	Proportion of body weight
Sensitivity	0.91 (0.76, 0.98)	0.97 (0.84, 1.00)
Specificity	0.69 (0.48, 0.86)	0.65 (0.44, 0.83)
Positive predictive value	0.79 (0.63, 0.9)	0.78 (0.62, 0.89)
Negative predictive value	0.86 (0.64, 0.97)	0.94 (0.73, 1.00)
Accuracy	81% (69%, 90%)	83% (71%, 92%)
Diagnostic odds ratio	22.5 (5.27, 95.9)	60.4 (7.05, 517.9)
Number needed to diagnose	1.66 (1.19, 4.19)	1.60 (1.21, 3.50)

Figures in parentheses represent the 95% confidence interval.

## Discussion

The Mirels score is commonly used in clinical practice. However, it has several limitations. A Mirels score of nine has a high sensitivity and serves as an appropriate threshold to predict impending pathological fractures. Our study also found that a Mirels score of nine is the optimum threshold for predicting fracture. However, a score of nine has a specificity of 35%. If the Mirels score is used as a strict indicator for surgery, two thirds of patients will have unnecessary surgery^11^.

The components of the Mirels score are not clearly defined in the original paper^7^. Although the pain component is accepted to be subjective, there is no method to quantify pain. The remaining three components of the Mirels score are intended to be objective. However, the boundaries for the site component of the score are not defined. It can be difficult to decide if a lesion is “peritrochanteric” (score three) or “lower limb” (score two). The score doesn’t distinguish between different locations within the peritrochanteric region. However, there is an important biomechanical difference between lesions in the medial and lateral part of the greater trochanter. Medial lesions have lower stiffness values, lower strength and are more likely to fracture than lateral lesions^12^.

Regarding the size component of the Mirels score; there is no definition of what should be measured. This component was found to be the most variable of all individual components of the score^8^. Analysis of the inter-observer and intra-observer variability of the Mirels score has demonstrated limited reproducibility and repeatability. Low-scoring lesions have more variance in the total score than higher scoring lesions^8^.

There are other factors which affect fracture risk in patients with metastatic disease. Obesity increases the risk of hip fractures because 25-hydroxyvitamin is sequestered in adipose tissue. Therefore, the resultant high serum levels of parathyroid hormone are likely to weaken cortical bone and increase fracture risk^13^.

**Figure 5:**
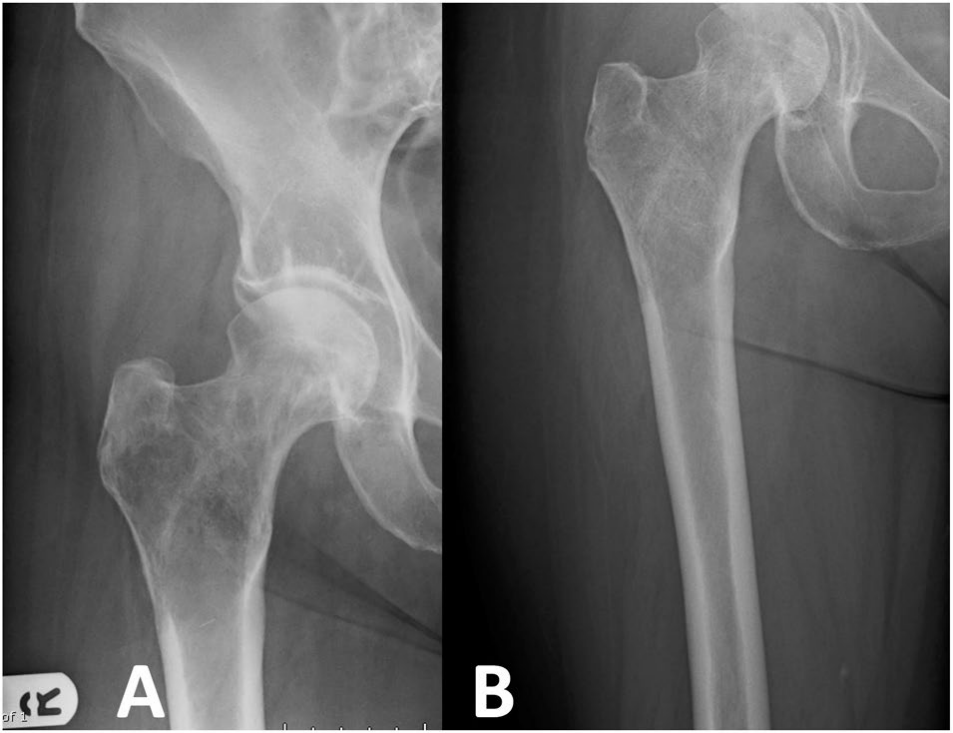
A patient at first presentation with metastatic breast cancer and a large lucent lesion in the peritrochanteric region and a Mirels score of ten (**A**). She was treated with denosumab, tamoxifen and radiotherapy. Follow up radiographs (**B**) showed the lesion had ossified and symptoms improved with medical management alone and surgical treatment was not required.

Mirels paper suggests surgical intervention once the threshold of nine is reached. However, in a previously undiagnosed breast or prostate malignancy, medical management can allow the bone to ossify without the need for surgical fixation (Fig. 5). Patients who can put more than 85% weight through the affected leg and have relatively mild symptoms, may not require immediate fixation and could be treated non operatively in first instance. These results show that there is a significant difference between the proportion of body weight patients with metastatic lower limb disease can put through their affected limb compared to the unaffected limb. Patients considered at high risk of fracture who underwent surgery could not put as much weight through their affected limb as those patients who were not offered surgery.

The ROC curves show a similar area under the curve for the Mirels score and the proportion of body weight the affected limb can bear. A threshold of 85% of total body weight through the affected limb has a similar sensitivity in predicting fracture than a Mirels score of nine. The proportion of body weight the affected leg can bear also has similar accuracy (83% vs 81%) and number needed to diagnose (1.66 vs 1.6) as the Mirels score. However, the diagnostic odds ratio is greater for the weight metric than the Mirels score (60.4 vs 22.5).

The current study is limited as the weight metric is only applicable to patients with metastatic disease affecting the lower limb. Whereas the Mirels score can also be used in the upper limb. Although it would be possible to extend the use of the weight metric to pelvic lesions, this has not been assessed or evaluated in this study.

Assessment will become difficult when a patient presents with metastatic disease in both legs as the proportion of body weight that can be put through each leg will become difficult to measure. Similarly, assessment may be difficult with coexisting spinal or other conditions to the legs.

Surgical intervention interferes with the natural history of disease and it is impossible to know if a bone would have fractured or not. All studies of this type are subject to this limitation and it is difficult to address.

It is accepted that the classification by the senior authors in ‘high-risk’ and ‘low-risk’ is subjective. However, this is difficult to avoid as it is opinion based. The decision to operate is not necessarily the same as this is also based on medical fitness and patient’s wishes. Furthermore, on occasion, it is appropriate to perform surgical treatment with intend to prolong the disease interval without impending skeletal failure.

The proportion of body weight that can be put through the affected leg may have measurement error. However, the variance of the proportion of body weight that could be put through the unaffected leg is small suggesting the measurement error to be unimportant.

This is a single institution study and external validation of our findings would be appropriate.

The proportion of body weight a patient can put through the affected limb seems an objective metric to predict fracture risk in patients with metastatic lower limb disease. It is suggested this metric measures pain and skeletal integrity indirectly. If a patient is unable to put 85% of their total body weight through the affected limb, treatment should be considered. The weight metric is simple to use in clinical practice and could be used in conjunction with the Mirels score to predict fracture risk in patients with metastatic disease of the lower limb.

## Supplementary information

Dataset 1

## Data Availability

All data generated or analysed during this study are included in this published article (and its Supplementary Information Files).
